# Caesarean scar choriocarcinoma: a case report and review of the literature

**DOI:** 10.1186/2047-783X-19-25

**Published:** 2014-05-14

**Authors:** Zhi-Da Qian, Xiao-Ming Zhu

**Affiliations:** 1 Department of Obstetrics and Gynecology, Women’s Hospital, School of Medicine, Zhejiang University, 1 Xueshi Road, Hangzhou, Zhejiang Province 310006, People’s Republic of China

**Keywords:** Caesarean scar, Choriocarcinoma, Treatment

## Abstract

**Objective:**

To report the clinical characteristics, pathologic findings and treatments of a patient with a Caesarean scar choriocarcinoma.

**Patient history:**

A 22-year-old woman had a diagnosis of primary gestational choriocarcinoma in a uterine Caesarean scar misdiagnosed as a normal Caesarean scar pregnancy. The patient underwent selective uterine artery embolization coupled with methotrexate arterial injection, along with dilatation and curettage of the uterine Caesarean scar. Finally, she received eight courses of multiagent chemotherapy. The reproductive function of the patient was preserved.

**Conclusions:**

Primary gestational choriocarcinoma out of the uterine corpus is a rare disease. A Caesarean scar choriocarcinoma is an extremely unusual example of this entity because of its unique position. To the best of our knowledge, this is the first report of this phenomenon. Our experience and a literature review suggest that a clinical diagnosis of a primary gestational choriocarcinoma of the uterine Caesarean scar is difficult to make, and uterine artery embolization is beneficial to prevent massive bleeding before curettage.

## Background

Gestational trophoblastic disease (GTD) includes the tumour spectrum of hydatidiform mole (complete and partial), invasive mole, choriocarcinoma and placental-site trophoblastic tumour. Gestational choriocarcinoma usually arises in the uterine body. It is a highly chemosensitive tumour type and has a very good prognosis, even in advanced stages. An accurate and prompt diagnosis is crucial. Extrauterine choriocarcinoma is a rare entity. Only a few cases have been reported in the literature up to now, and most of these cases were located in the uterine cervix [1-4]. Other extrauterine locations have also been reported, including the ovary [5,6], Fallopian tube [7], vagina [8], vulva [9] and gut [10]. Choriocarcinoma in a Caesarean scar has not been reported before. Here, we present our experience with the diagnosis and management of a rare case of a Caesarean scar choriocarcinoma.

## Case presentation

**Figure 1:**
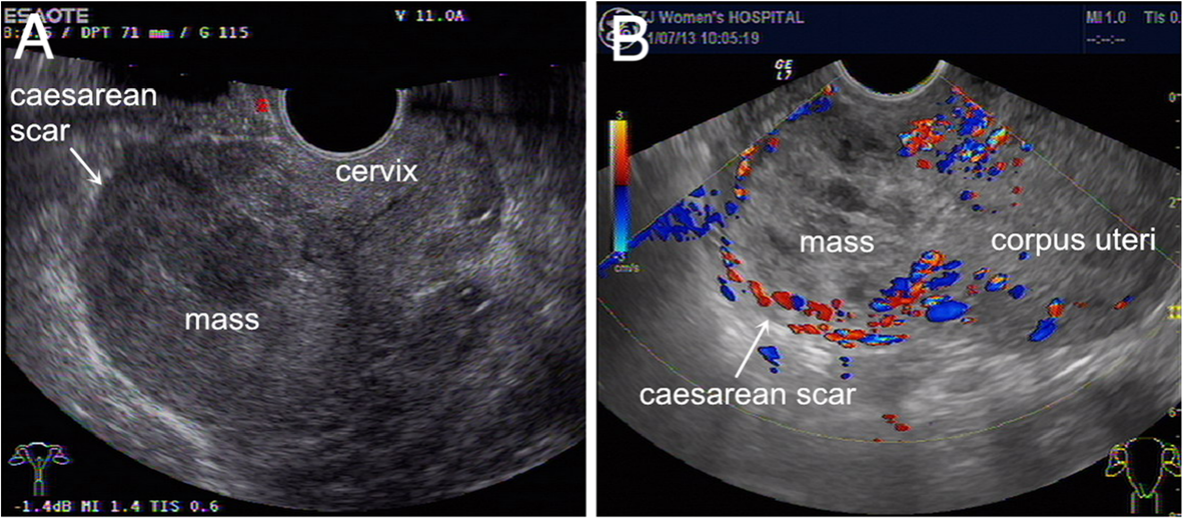
**Transvaginal ultrasonography of the patient before (A) and after (B) dilation and curettage (D&C).** The uterine cavity and cervical canal were empty. A mass implanted in the anterior wall of the uterine Caesarean scar (indicated by the arrow) embedded and surrounded by thin myometrium and separated from the endometrial cavity was visible on the retroverted uterus. Ultrasonography revealed that the mass was heterogeneous with a mixture of cystic and solid echogenicity. **(A)** Two days before D&C (longitudinal section). The size of the mass was 6.6 × 5.6 × 5.5 cm. RI = 0.38. **(B)** Five days after D&C (longitudinal section). The size of the mass was 4.7 × 5.7 × 4.5 cm. Abundant blood flow signals and low RI around the mass, RI = 0.29.

On 6 July 2011, a 22-year-old woman (gravida 4, para 2) was admitted to our hospital with a complaint of amenorrhea for 47 days and irregular vaginal bleeding for half a month. She had had her first pregnancy three years previously, which ended with a full-term vaginal delivery. Her second normal pregnancy ended in an induced abortion in the first trimester two years previously. Her third pregnancy ended in a full-term delivery by Caesarean section in July 2010. She had an inevitable abortion ending in curettage four months previously. Her menstrual cycle was regular (30 days) with seven days duration. Her most recent menstrual period was on 19 May 2011. Bimanual examination revealed a mildly enlarged uterine corpus with an obviously enlarged uterine isthmus and a closed cervical os. A transvaginal sonogram showed a 6.6 × 5.6 × 5.5 cm mass implanted in the anterior wall of the uterine isthmus embedded in and surrounded by myometrium and separated from the endometrial cavity. The lesion was bulging toward the serosa with a thin layer of overlying myometrium (Figure 1A). Both the uterine cavity and the cervical canal were empty. Pulsed Doppler ultrasonography showed abundant blood flow signals and a low resistive index (RI = 0.38) around the lesion. The results of computed tomography (CT) of the chest and brain were normal. There were no pathologic findings in the upper abdominal ultrasound. The blood level of β-human chorionic gonadotropin (β-HCG) was 312,468 IU/l (normal value, <5.3 IU/l) on 6 July 2011.

Because Caesarean scar choriocarcinoma is rare, its clinical diagnosis is very hard to make in a patient without metastasis before a pathological examination. Furthermore, we had no experience of the clinical characteristics and diagnosis of Caesarean scar choriocarcinoma. The patient was diagnosed as having a normal Caesarean scar pregnancy (CSP). After written informed consent was obtained, she was treated with a selective uterine artery embolization (UAE) coupled with an arterial injection of 70 mg of methotrexate (MTX) after a routine clinical and laboratory evaluation on 7 July 2011. The patient underwent careful dilation and curettage (D&C) under transabdominal ultrasound guidance the next day. Bleeding was initially brisk but decreased substantially by the time most adherent tissue had been removed. The curettage specimen consisted of approximately 4.0 × 4.0 × 3.0 cm of necrotic tissue mixed with blood clots. An unusually large anterior uterine wall defect was found on the ultrasonic scan during the operation. The total blood loss was 200 ml and iodoform gauze packing was left *in situ* for 24 hours to decrease the risk of heavy vaginal bleeding. The patient’s hemodynamic status remained stable during and after the procedure. The postoperative recovery was uneventful, and the patient’s serum β-HCG levels declined from 189,930 IU/l to 110,984 IU/l the day after the operation.

**Figure 2:**
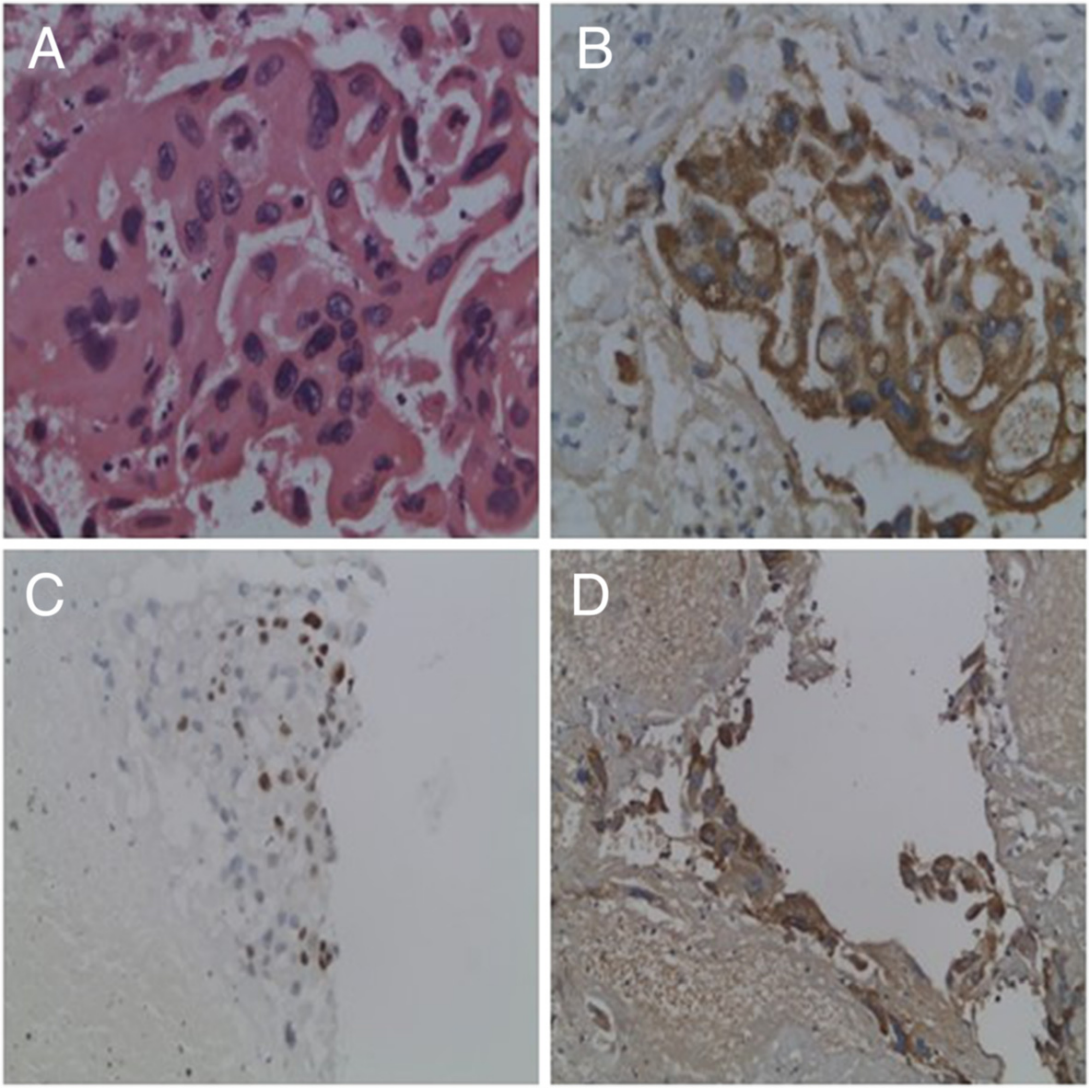
**Histologic section of Caesarean scar choriocarcinoma. (A)** Marked nuclear and cellular atypia and increased mitotic activity (H&E, ×400). **(B)** Immunohistochemical staining of tumour cells was positive for β-HCG (immunohistochemistry, ×400). **(C)** Ki-67 positive tumour cells (immunohistochemistry, ×200). **(D)** Human placental lactogen positive tumour cells (immunohistochemistry, ×200).

However, transvaginal ultrasonography revealed a 4.7 × 5.7 × 4.5 cm complex mass protruding into the bladder and increased vascularity at the anterior lower uterine wall (RI = 0.29) five days after the D&C (Figure 1B). A histological examination confirmed the diagnosis of choriocarcinoma. Histology revealed a proliferation of trophoblastic and syncytiotrophoblastic cells with clearly malignant features in the clot, and chorionic villi were not identified (Figure 2A). Immunohistochemical staining for β-HCG was strongly positive (Figure 2B), and the Ki-67 index was high in the tumour tissue (Figure 2C). Human placental lactogen was weakly positive in the tumour tissue (Figure 2D). The diagnosis of a Caesarean scar choriocarcinoma was confirmed on the basis of all the findings. The patient was accepted as International Federation of Gynecology and Obstetrics stage I:8 and received eight courses of multiagent chemotherapy (etoposide, actinomycin D, methotrexate, cyclophosphamide and vincristine, EMA/CO). Her progress was monitored with serial weekly blood β-HCG measurements. The β-HCG levels declined from 18,121 IU/l before the first course of EMA/CO therapy to 3.57 IU/l after four cycles of EMA/CO therapy on 8 September 2011. The post-chemotherapy period was excellent, without any major complications.

## Discussion

Gestational choriocarcinoma may accompany or follow any type of pregnancy, such as a hydatidiform mole, a normal term pregnancy, an abortion or an ectopic pregnancy. A CSP is defined as an ectopic pregnancy embedded in the myometrium of a previous Caesarean scar, which is a late serious complication of a Caesarean section. The incidence of CSP is 1:2,216 and its rate is 6.1% in women with an ectopic pregnancy and at least one previous Caesarean section [11]. The incidence of CSP is extremely low. However, it has been increasing with the recently increasing numbers of Caesarean sections. Early diagnosis and early treatment remain the key for the successful treatment of CSP. Colour Doppler ultrasound is important in its early diagnosis and treatment. Different therapeutic modalities may be selected according to specific patient conditions [12].

We performed a PubMed search from January 1960 to October 2013 using the keywords ‘Caesarean scar’ , ‘choriocarcinoma’ , ‘molar’ and ‘hydatidiform’ to look for reports of Caesarean scar GTD. We could not find any published cases of Caesarean scar choriocarcinoma. So far, three cases of Caesarean scar molar pregnancies have been reported in the literature [13-15], and as with the rare choriocarcinoma in a Caesarean scar, they presumably carry a high risk of uterine rupture and uncontrollable haemorrhage. A prompt and accurate diagnosis is crucial. However, primary gestational choriocarcinoma out of the uterine cavity is a very rare disease. Caesarean scar choriocarcinoma is an extremely unusual example of this entity because of its unique position. Awareness of the possibility of lesions in a previous Caesarean scar is needed to avoid potentially catastrophic complications.

In this report, the patient’s serum β-HCG level (312,468 IU/l) was much higher than in a normal pregnancy (50,000 to 100,000 IU/l). Furthermore, the patient complained of amenorrhea for 47 days and had an obvious, enlarged uterine isthmus mass (6.6 × 5.6 × 5.5 cm) and abundant blood flow. A high degree of suspicion is essential for an early diagnosis of Caesarean scar GTD. Unfortunately, this case was not correctly diagnosed before treatment. Caesarean scar choriocarcinoma should be included in the differential diagnosis of cervical lesions in patients in their reproductive years. It is easily misdiagnosed as a uterine cervical pregnancy, a threatened abortion, a normal CSP, a cervical polyp or another cervix neoplasm. The clinical diagnosis of primary gestational choriocarcinoma in a Caesarean scar is very difficult to make before a pathological examination. Wu *et al.*[13] reported a case of a partial molar pregnancy in a Caesarean scar that had been misdiagnosed as a threatened abortion at a local medical clinic in 2006. Michener *et al.*[14] reported a second case of a Caesarean scar molar pregnancy, and the diagnosis was delayed until 10 months later when the patient presented with vaginal haemorrhage, which required an emergency hysterectomy. The histological examination confirmed molar tissue in the hysterectomy specimen. Abnormal elevated serum β-HCG levels coupled with CT scans of the chest and brain are helpful to establish a diagnosis. When ultrasound findings indicate a suspected CSP, abnormally elevated serum β-HCG levels could increase the suspicion of a GTD in the Caesarean scar, especially choriocarcinoma. Clinicians should be aware of this diagnosis in spite of a lack of metastasis.

A suction evacuation under ultrasound guidance was needed to obtain tissue for histological diagnosis. In our case, the patient had a blood loss of 200 ml during the D&C. The operation was performed 24 hours after a UAE to reduce the risk of haemorrhage. Ko *et al.*[15] reported a case of a Caesarean scar molar pregnancy diagnosed before treatment. Active bleeding was present at the end of the D&C, and a UAE was performed to control the bleeding immediately. However, the total blood loss was 1,000 ml. Therefore, should UAE be offered to all patients with Caesarean scar GTD before suction curettage?

Uterine artery embolization is a minimally invasive nonsurgical treatment widely used to control haemorrhage and preserve the uterus and the patient’s future fertility. It is an alternative to treatments for CSP, and it has been shown to have a high success rate and a low complication rate. Followed by uterine curettage, UAE might be an effective and safe treatment for CSP [16]. Superselective embolization of both uterine arteries was performed using gelatin sponge powder by two experienced radiologists. The Seldinger technique was applied to puncture and catheterize the bilateral internal iliac arteries via the right femoral artery, and the procedure was performed under local anaesthesia. Postembolization angiography was performed to confirm that the occlusion of the vessels was complete. Uterine artery embolization is a priority alternative for the D&C of GTD within Caesarean scars for several reasons. Serious complications related to UAE have been reported [17]. This includes labial or vaginal necrosis with bladder fistula, endometrial atrophy or permanent amenorrhea. There is no clear consensus on the optimal management of CSP; UAE followed by suction curettage appears to have more of an advantage and might be a priority option. Uterine artery embolization prevents massive bleeding and preserves the uterus, so we recommend UAE as a priority alternative for the D&C of GTD within Caesarean scars for several reasons. First, patients with this pathology are at a high risk of severe, potentially life-threatening bleeding, which may lead to a hysterectomy, with dramatic consequences for their reproductive future. The purpose of UAE is to block the blood flow in the designated uterine artery to decrease vascularization at the site of the lesion. Second, UAE can reduce the occurrence of patients with an unstable hemodynamic status owing to heavy bleeding. Finally, UAE makes the D&C procedure safer and more proactive.

This case was treated with preventive UAE coupled with a MTX arterial injection before the D&C. Methotrexate is an agonist of folinic acid implicated in DNA synthesis. A combined MTX regimen, which may be systemic or local, single dose or multidose, can interrupt CSPs. Pascual *et al.*[18] reported that a patient with CSP had been treated conservatively and successfully with a local injection of MTX into the gestational sac under transvaginal ultrasonographic guidance.

Wang *et al.*[19] reported that 15 out of 128 CSP patients still had massive bleeding (blood loss of 500 ml or more) during D&C after UAE. They found that a gestational age of eight weeks or more, a CSP mass diameter of 6 cm or more, and a thinner myometrium at the implantation site were risk factors for massive bleeding during surgery after a preventive UAE. This patient had a large amount of gestational tissue in the Caesarean scar and a thinner myometrium at the implantation site. However, the intraoperative blood loss was 200 ml in this case. This might be because the main purpose of the D&C was to obtain tissue for a histological diagnosis. Ultrasonography revealed there was still a 4.7 × 5.7 × 4.5 cm mass at the anterior lower uterine wall five days after the D&C. This patient might have had massive bleeding if we had tried to remove the mass completely. To prevent massive bleeding, we suggest that residual gestational tissue might not be removed completely when tissue is closely attached to the uterus, even after a preventive UAE.

## Conclusions

In conclusion, owing to the increasing incidence of CSP, doctors may encounter more cases of Caesarean scar GTD. Because Caesarean scar choriocarcinoma is rare, no therapeutic protocols have been established. There may be different treatment options for different patients. We believe that the treatment selection should be based on the characteristics of the patient, combining the therapies of choriocarcinoma with CSP. The conservation of reproductive function should be considered if possible, and preventive UAE is beneficial to prevent massive haemorrhage before careful D&C. However, further studies are needed to reach a more definitive conclusion.

## Consent

Written informed consent was obtained from the patient for publication of this case report and any accompanying images. A copy of the written consent is available for review by the editor in chief of this journal.

## Abbreviations

β-HCG: β-human chorionic gonadotropin; CSP: Caesarean scarpregnancy; CT: computed tomography; D&C: dilation and curettage; EMA/CO: etoposide, actinomycin D, methotrexate, cyclophosphamide and vincristine; GTD: gestational trophoblastic disease; H&E: haematoxylin and eosin; MTX: methotrexate; RI: resistive index; UAE: uterine artery embolization.

## Competing interests

The authors declare that they have no competing interests.

## Authors’ contributions

ZDQ and XMZ collected the case information and drafted the manuscript. Both authors read and approved the final manuscript.
